# The impact of the time factors on the exercise-based cardiac rehabilitation outcomes of the patients with acute myocardial infarction after percutaneous coronary intervention: a systematic review and meta-analysis

**DOI:** 10.1186/s12872-023-03692-z

**Published:** 2024-01-06

**Authors:** Peiyu Zhang, Chaofeng Niu, Lijing Zhang, Haixia Lai, Birong Liu, Diyang Lv, Rui Zhuang, Yong Liu, Di Xiao, Liyong Ma, Meng Li

**Affiliations:** 1 Department of Cardiology, Dongzhimen Hospital, Beijing University of Chinese Medicine, Beijing, China; 2 Department of Cardiology, Dongfang Hospital, Beijing University of Chinese Medicine, Beijing, China

**Keywords:** Cardiac rehabilitation, Time, Acute myocardial infarction, Percutaneous coronary intervention, Meta-analysis

## Abstract

**Background and objective:**

Cardiac rehabilitation (CR) has been demonstrated to improve outcomes in patients with acute myocardial infarction (AMI) after percutaneous coronary intervention (PCI). However, the optimal CR initiation time and duration remain to be determined. This study aimed to explore the impact of the time factors on the CR outcomes in AMI patients who received PCI by the method of meta-regression analysis.

**Methods:**

We searched five databases (PubMed, Embase, Cochrane Library, Web of Science and Google scholar) up to October 31, 2023. Meta-regression analysis was utilized to explore the impact of the time factors on the effect sizes. Subgroups with more than 3 studies were used for meta-regression analysis.

**Results:**

Our analysis included 16 studies and a total of 1810 patients. The meta-regression analysis revealed that the initiation time and duration of CR had no significant impact on the occurrence of arrhythmia, coronary artery restenosis and angina pectoris. The initiation time and duration of CR also had no significant impact on the changes in left ventricular ejection fraction (LVEF, starting time: estimate = 0.160, *p* = 0.130; intervention time: estimate = 0.017, *p* = 0.149), left ventricular end-diastolic volume (LVEDV, starting time: estimate = − 0.191, *p* = 0.732; intervention time: estimate = − 0.033, *p* = 0.160), left ventricular end-systolic volume (LVESV, starting time: estimate = − 0.301, *p* = 0.464; intervention time: estimate = 0.015, *p* = 0.368) and 6-minute walk test (6MWT, starting time: estimate = − 0.108, *p* = 0.467; intervention time: estimate = 0.019, *p* = 0.116).

**Conclusion:**

Implementation of CR following PCI in patients with AMI is beneficial. However, in AMI patients, there is no significant difference in the improvement of CR outcomes based on different CR starting times within 1 month after PCI or different durations of the CR programs. It indicates that it is feasible for patients with AMI to commence CR within 1 month after PCI and continue long-term CR, but the time factors which impact CR are intricate and further clinical research is still needed to determine the optimal initiation time and duration of CR.

**Supplementary Information:**

The online version contains supplementary material available at 10.1186/s12872-023-03692-z.

## Introduction

Acute myocardial infarction (AMI) is one of the important causes of cardiovascular death [1]. Percutaneous coronary intervention (PCI) is crucial in reconstructing coronary arterial revascularization [2]. Cardiac rehabilitation (CR) is a well-established and scientifically proven approach that incorporates patient education, behavior modification techniques, and exercise training to significantly improve secondary prevention outcomes in patients diagnosed with cardiovascular disease [3]. The safety of early CR after PCI has been identified [4]. Engagement in CR post-PCI has been linked to a noteworthy decrease in both all-cause mortality (ACM) and cardiovascular mortality [5]. There is currently no agreement on the optimal time to begin CR following PCI in patients with AMI [6]. An earlier study indicated that rehabilitation exercises should commence approximately 6 weeks after a cardiovascular event to allow sufficient time for medical stabilization and recovery [7]. It is common for CR and secondary prevention programs to start no earlier than 4–6 weeks after a patient has been discharged from the hospital [8]. According to the 2014 AHA/ACC guidelines for Non-ST-Elevation acute coronary syndrome (ACS), in patients treated with PCI, aerobic exercise training is generally recommended to commence within 1 to 2 weeks after discharge [2].

In recent years, a growing body of research has investigated the potential advantages of starting CR at an earlier stage [4, 9–11]. Patients who suffered from AMI and started engaging in progressive exercise within 1 week after undergoing PCI treatment demonstrated significantly higher left ventricular ejection fraction (LVEF) and quality of life scores compared to the control group, according to a study conducted over a 6-month period [12]. A 6-month CR program initiated 3–7 days after PCI in AMI patients can improve LVEF and prevent ventricular remodeling [13].

It is worth noting that some studies have also supported initiating CR in patients with AMI after PCI and discharge from the hospital. Xiao ML et al.’s study revealed that a 12-month community-based physical rehabilitation significantly reduced the risk of major adverse cardiovascular events (MACE) and improved cardiac function and physical endurance in patients who received PCI following AMI [14]. Zhang Y et al.’s study also supported that community-based CR starting on the second week after discharge and lasting for 6 months can lead to significant improvements in cardiac ejection fraction, exercise tolerance, and cardiovascular risk factors reduction among patients with AMI [15]. Several meta-analyses examining the effectiveness of CR have consistently demonstrated that it can help reduce the incidence of cardiovascular events [16–18]. There were few studies directly exploring the impact of different initiation times or durations of CR programs on CR outcomes. Therefore, we conducted this meta-analysis to explore the impact of the time factors on CR outcomes in patients with AMI who underwent PCI.

## Method

We performed this study based on the latest Preferred Reporting Items for Systematic reviews and Meta-Analyses (PRISMA) guidelines [19]. This study has been registered in PROSPERO (CRD42023399979). Our analysis is based on prior research and does not require ethical approval or informed consent from patients.

### Search strategy

We systematically searched the following databases (up to October 31, 2023): PubMed, Embase, Cochrane Library, Web of Science and Google scholar. The search terms included “cardiac rehabilitation”, “exercise”, “training”, “percutaneous coronary intervention” and “acute myocardial infarction”. Additional details about the retrieval strategies can be found in S_Table 1.

### Inclusion and exclusion criteria

Inclusion criteria: 1) Randomized controlled trials (RCTs) or observational studies investigating the efficacy of CR in patients with AMI after PCI; 2) Studies comparing exercise versus no exercise (usual care); 3) Measurement outcomes include cardiovascular events, echocardiography parameters, 6-minute walk test (6MWT), heart rate, blood pressure and blood fat; 4) Articles published in English. Exclusion criteria: 1) Studies did not include patients who underwent PCI treatment for AMI or included patients who underwent PCI treatment but did not meet the diagnostic criteria for AMI; 2) Studies comparing the exercise group and non-exercise group was not conducted; 3) The studies’ reports were incomplete or had no results; 4) Reviews, meta-analyses, protocols, letters, case reports and animal studies.

### Quality assessment

To evaluate the quality of the included study, we used the Cochrane Collaboration’s tool for assessing the risk of bias in RCTs [20]. The assessing tool included 7 items: random sequence generation (selection bias), allocation concealment (selection bias), blinding of participants and personnel (performance bias), blinding of outcome assessment (detection bias), incomplete outcome data (attrition bias), selective reporting (reporting bias) and other biases. The observational studies were assessed for quality using the Newcastle-Ottawa Scale (NOS) [21], which includes six items: “is the case definition adequate”, “representativeness of the cases”, “details of methods of assessment”, “blind methods were used in the measurement”, “comparability of cases and controls was described”, and “explain how missing data where addressed”. Each item was classified into three grades, including “low risk”, “unclear risk” and “high risk”. The quality assessment was conducted by two separate authors. Whenever there was any disagreement between the two authors, they would consult with the third author to reach a consensus.

### Data extraction

Two authors extracted the included study’s first author, publication year, patient characteristics, intervention measures, starting time of intervention and duration of intervention, as well as primary outcome indicators. Any disagreements were resolved through discussion with another author until a consensus was reached. We contacted the original authors to obtain more complete data.

### Statistical method

The net effect sizes were calculated by Qiao H et al.’s online tools [22]. For categorical variables, the combined results were reported as Risk Ratio (RR) and 95% confidence interval (CI). The continuous variables were converted into standardized mean difference (SMD) and 95% CI for analysis. All analyses were conducted by R software (version 4.2.3). To conduct our meta-analysis, we primarily used “metafor” package (version 4.0.0) of R software [23]. A *p*-value of less than 0.05 was considered statistically significant. Meta-regression analysis was utilized to explore the impact of the time factors on the effect sizes. Subgroups with more than 3 studies were used for meta-regression analysis. Funnel plot was used to show publication bias.

## Result

### Study and patient characteristics

**Fig. 1:**
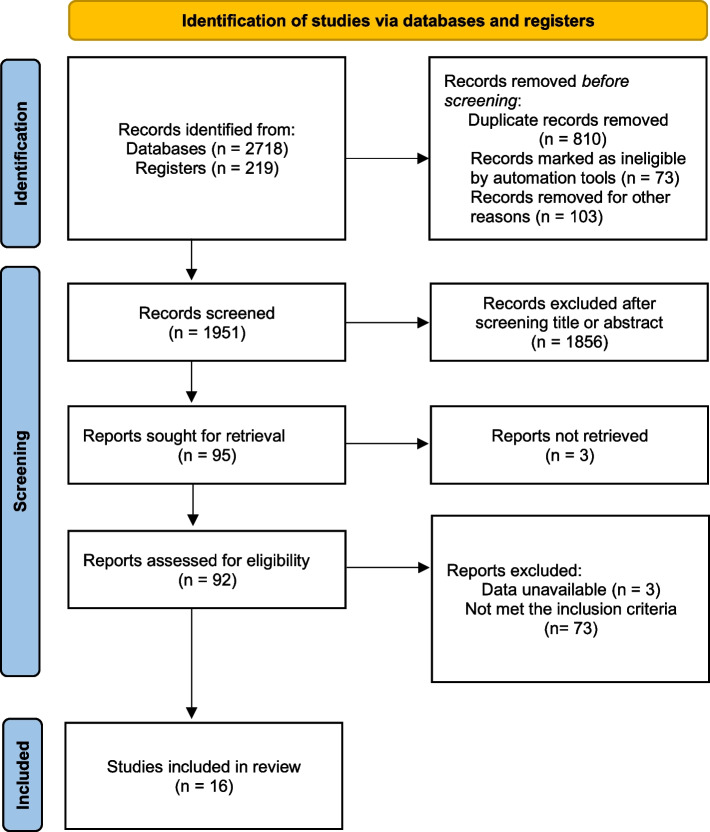
Flow chart of study screening

Table 1 presents the characteristics of the 16 studies included in this analysis. These studies were published in English between 2003 and 2023, with 11 studies conducted in China [12–15, 24–30] and one study each conducted in Japan [31], Korea [32], Brazil [33], Iran [34], and Pakistan [35]. 10 studies were RCTs [12–15, 24, 25, 29, 31, 33, 34], 2 studies were retrospective cohort studies [26, 28], and 4 study was a prospective cohort study [27, 30, 32, 35]. The included studies involved a total of 1810 patients (774 patients in the rehabilitation group and1036 patients in the control group). The average age of the patients was 60.3 ± 10.32. CR programs were mainly based on supervised aerobic exercise. Among the studies we included, the CR program started as early as the second day after PCI (0.1 weeks) and as late as 1 month after PCI. The shortest intervention duration is 1 week, and the longest is 2 years. The frequency of exercise mostly ranged from 3 to 5 times per week, with each session lasting between 30 to 60 minutes. The process of study screening is depicted in Fig. 1.

**Table 1:** The characteristics of the included studies

Author	Year	Country	Gender (M/F)	Age	Type	Group (RG/CG)	Starting Time (weeks after PCI)	Intervention Time (weeks)	Intervention	Frequency
Koizumi T et al.	2003	Japan	RG (13/1) CG (13/2)	56.6 ± 10.67	RCT	14/15	4	12	walking	> 30 minutes per day
Zheng H et al.	2008	China	NA	NA	RCT	27/30	1	24	bicycle ergometer	3 times per week, 1 hour each time
Peixoto TC et al.	2015	Brazil	RG (33/12) CG (29/14)	56.4 ± 10.2	RCT	45/43	0.1	4	walking	4 times per week, 25–45 minutes each time
Xu L et al.	2016	China	RG (22/4) CG (22/4)	55.7 ± 9.2	RCT	26/26	0.1	5	Walking, jogging	3 times per day, 15–30 minutes each time
Abtahi F et al.	2017	Iran	RG (14/11) CG (15/10)	53.7 ± 6.9	RCT	25/25	1	8	Aerobic exercise training	3 times per week, 1 hour each time
Zhang Y et al.	2018	China	RG (59/6) CG (54/11)	70 ± 10.5	RCT	65/65	3	20	Walking	2–5 times per week, 15–45 minutes each time
Chen MG et al.	2020	China	RG (29/14) CG (30/9)	60.7 ± 11.1	RCT	43/39	0.1	24	Baduanjin Sequential Therapy	5 times per week, 30 minutes each time
Jiang MH et al.	2021	China	RG (31/18) CG (33/16)	59.2 ± 9.13	RCT	49/49	0.1	24	Progressive exercise of kinetic energy	3 times per day, 5 minutes each time (the frequency gradually increased)
Xiao ML et al.	2021	China	RG (61/21) CG (64/18)	59.5 ± 9.0	RCT	82/82	1	48	Walking, bicycling	3–5 times per week, 50–60 minutes each time
Cai TT et al.	2023	China	RG (12/37) CG (10/39)	57.6 ± 2.51	RCT	49/49	0.1	24	A step-by-step activity process, Walking, bicycling	2 times per day, 15–30 minutes each time
Lee HY et al.	2013	Korea	RG (30/7) CG (31/6)	59.6 ± 9.77	Prospective cohort study	37/37	4	42	Treadmill, bicycle ergometer	3 times per week, 1 hour each time
Yu HQ et al.	2021	China	RG (41/17) CG (39/18)	60.2 ± 2.5	Retrospective cohort study	58/57	0.1	12	Walking, jogging	3 times per day, 10–30 minutes each time
Ma JR et al.	2021	China	RG (90/14) CG (274/95)	62.3 ± 12.2	Prospective cohort study	104/369	1.5	24	Aerobic exercise	3–5 times per week, 40–110 minutes each time
Peng XM et al.	2022	China	RG (41/9) CG (39/11)	55.2 ± 7.98	Retrospective cohort study	50/50	0.1	1	Walking	2 times per day, 15–30 minutes each time
Shah ZA et al.	2022	Pakistan	RG (34/6) CG (30/10)	67 ± 6.8	Prospective cohort study	40/40	4	12	Walking, brisk walking	At least 15 to 30 minutes daily
Huang CP et al.	2023	China	RG (32/28) CG (33/27)	55.3 ± 6.08	Prospective cohort study	60/60	0.1	24	Progressive exercise of kinetic energy	NA

M/F, male/female; NA, not available; RCT, randomized controlled trial; RG/CG, rehabilitation group/control group

### Quality assessment

Four RCTs explicitly mentioned allocation concealment [24, 25, 33, 34]. Only one RCT employed the single-blind method [13]. Four RCTs did not used blind methods for outcome measurement and we labeled them as high risk [12, 14, 15, 29]. Among the observational studies, only one study used blind methods to measure the outcomes [32]. There was a significant difference in the number of participants between the experimental group and the control group in an observational study and we believed that comparability poses a high risk [27]. In short, most of the included studies’ quality were moderate (S_Fig. 1).

### Heterogeneity

These studies had significant variations in methodology and statistical approaches. The result of LVEF Meta-analysis showed high heterogeneity (RCT: I^2^ = 80%, tau^2^ = 0.195, *p* < 0.01; observational study: I^2^ = 88%, tau^2^ = 0.270, p < 0.01). Therefore, their effect sizes cannot be pooled directly [36]. We performed meta-regression analysis on the outcome indicators of the included studies, incorporating rehabilitation starting time and duration as influential factors.

### Cardiovascular events

**Fig. 2:**
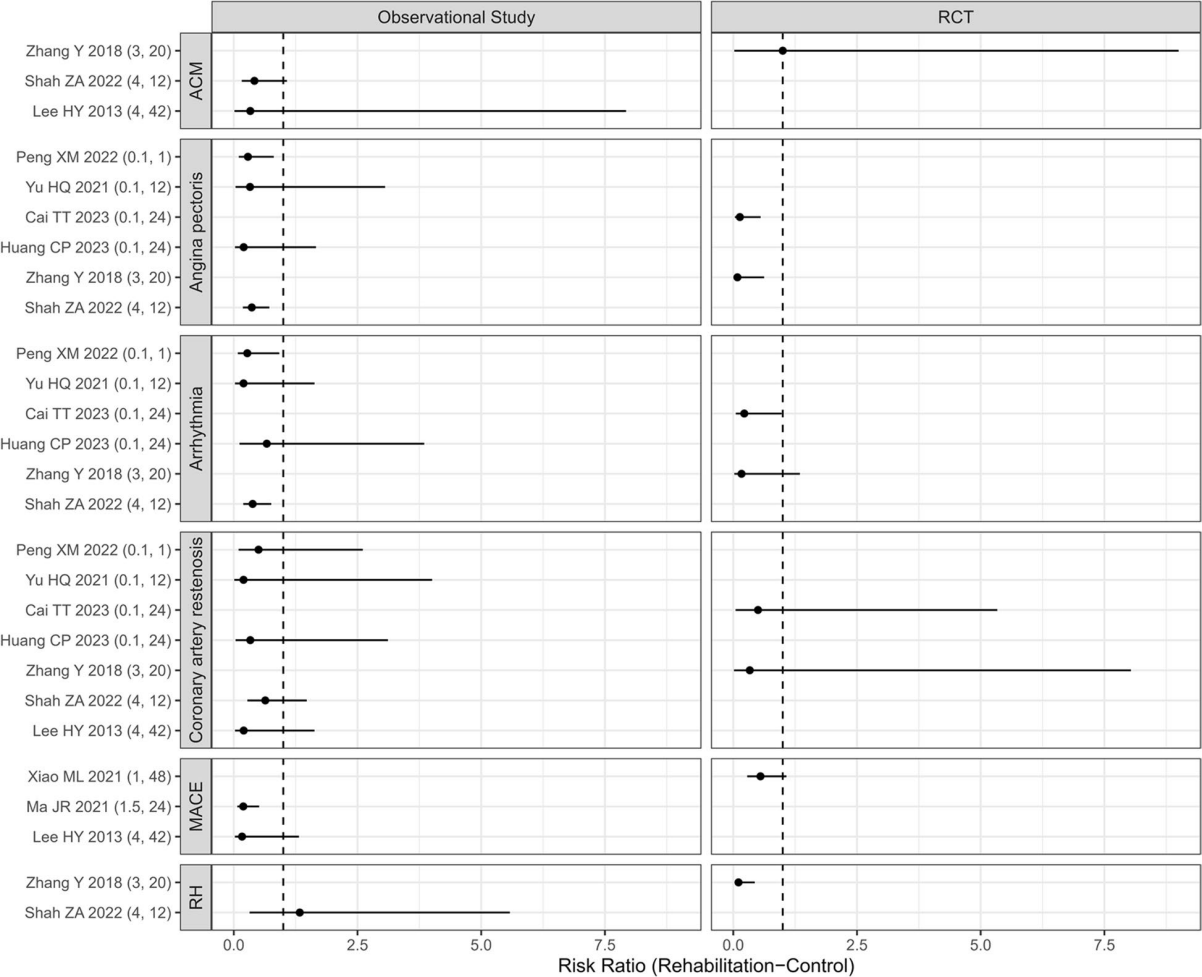
The forest plot of the Risk Ratio of cardiovascular events. ACM, All-cause mortality; RH, Rehospitalization

Nine studies reported the cardiovascular events during the intervention (3 RCTs [14, 15, 29] and 6 observational studies [26–28, 30, 32, 35]). The meta-regression analysis revealed that the initiation time and duration of CR had no significant impact on the occurrence of three types of cardiovascular events (arrhythmia, coronary artery restenosis and angina pectoris, S_Table 2 and Fig. 2).

### Echocardiography parameters

**Fig. 3:**
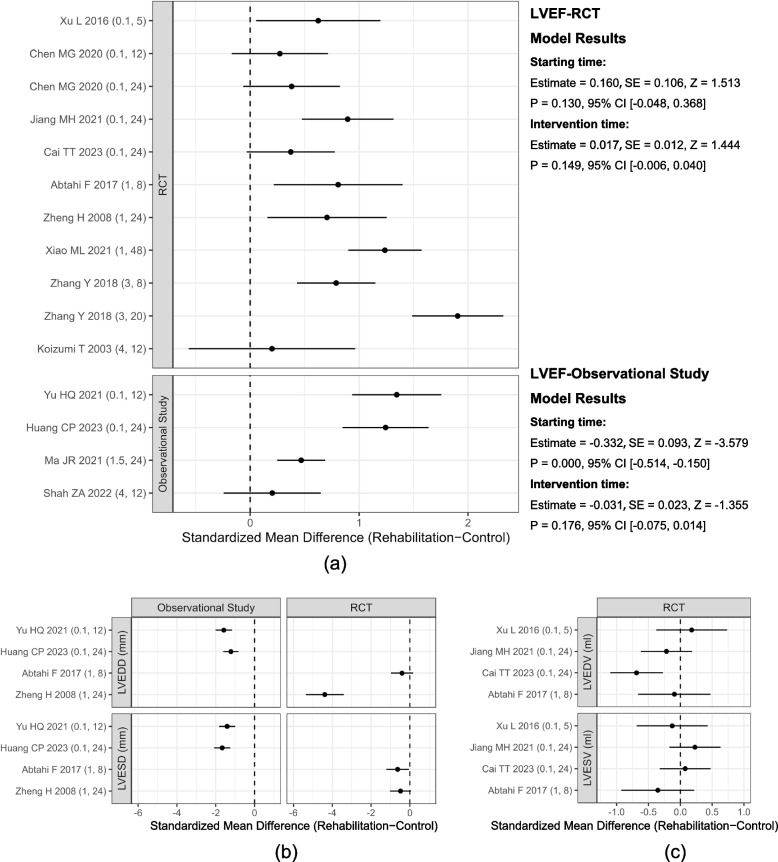
Forest plot of the changes in echocardiography parameters. **a** LVEF; **b** LVEDD and LVESD; **c** LVEDV and LVESV

Thirteen studies reported the mean and standard deviation (SD) of LVEF (9 RCTs [12–15, 24, 25, 29, 31, 34] and 4 observational studies [26, 27, 30, 35]), 4 studies (2RCTs [13, 34] and 2 observational studies [26, 30]) reported left ventricular end-diastolic diameter (LVEDD) and left ventricular end-systolic diameter (LVESD), 4 RCTs [12, 24, 29, 34] reported left ventricular end-diastolic volume (LVEDV) and left ventricular end-systolic volume (LVESV). The mean and SD of the changes in echocardiography parameters before and after intervention in both rehabilitation and control groups were calculated on an online platform [22]. In the meta-regression analysis of observational studies, the starting time demonstrated a significant impact on the changes in LVEF (starting time: estimate = − 0.332, *p* = 0.0003, S_Table 2 and Fig. 3a), whereas the duration did not show any significant effect (intervention time: estimate = − 0.031, *p* = 0.176, S_Table 2 and Fig. 3a). However, the meta-regression analysis of RCTs indicated that the initiation time and duration of CR had no significant impact on the changes in LVEF (starting time: estimate = 0.160, *p* = 0.130; intervention time: estimate = 0.017, *p* = 0.149, S_Table 2 and Fig. 3a), LVEDV (starting time: estimate = − 0.191, *p* = 0.732; intervention time: estimate = − 0.033, *p* = 0.160, S_Table 2 and Fig. 3c), and LVESV (starting time: estimate = − 0.301, *p* = 0.464; intervention time: estimate = 0.015, *p* = 0.368, S_Table 2 and Fig. 3c).

### 6MWT

**Fig. 4:**
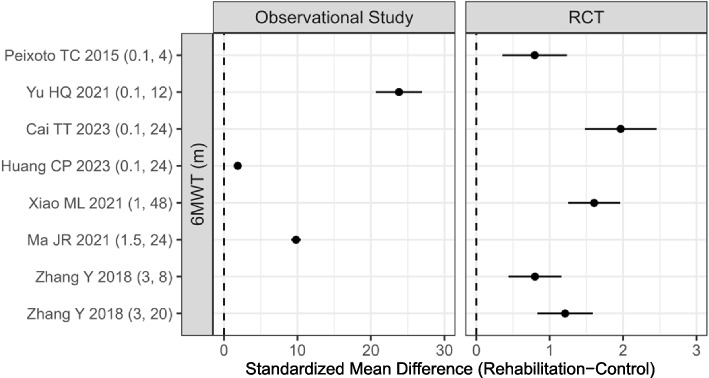
Forest plot of the changes in 6MWT

Seven studies reported the mean and SD of 6MWT before and after intervention in both rehabilitation and control groups (4 RCTs [14, 15, 29, 33] and 3 observational studies [26, 27, 30]). The results of the meta-regression analysis of RCTs did not reveal any significant association between changes in 6MWT and either initiation time or duration (starting time: estimate = − 0.108, *p* = 0.467; intervention time: estimate = 0.019, *p* = 0.116, S_Table 2 and Fig. 4).

### Cardiovascular risk factors

**Fig. 5:**
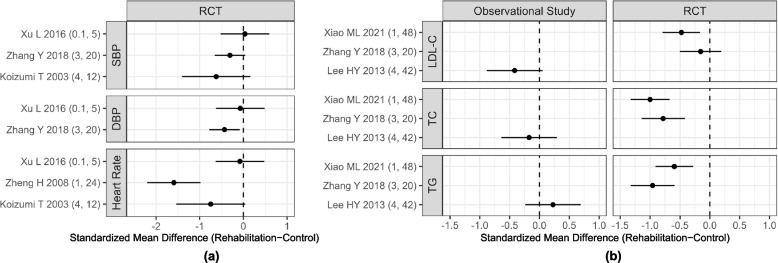
Forest plot of the changes in cardiovascular risk factors. **a** Blood pressure and heart rate; **b** Blood lipids (LDL-C, TC and TG)

Three RCTs reported the mean and SD of heart rate before and after intervention [13, 24, 31], 3 RCTs reported blood pressure [15, 24, 31], and 3 studies (2 RCTs [14, 15] and 1 observational study [32]) reported total cholesterol (TC), triglyceride (TG), and low density lipoprotein cholesterol (LDL-C). The forest plots of these clinical data was shown in Fig. 5.

### Publication bias

We conducted publication bias testing on analyses that included more than 10 studies (S_Fig. 2). The funnel plots of the SMD for LVEF (RCTs) indicated the presence of possible publication bias.

## Discussion

A total of 16 studies, including 1810 participants (774 in the rehabilitation group and 1036 in the control group), were analyzed in our meta-analysis. Among the studies we included, the CR program started as early as the second day after PCI (0.1 weeks) and as late as 1 month after PCI. The shortest intervention duration is 1 week, and the longest is 2 years. The results of the meta-regression analysis showed that there was no significant difference in the improvement of CR outcomes among AMI patients when comparing different CR starting times within 1 month after PCI or different durations of the CR programs.

PCI is considered as one of the commonly employed methods for reperfusion therapy. The benefits of early CR after PCI, particularly in patients with AMI, are increasingly recognized. It is safe to start exercising early after coronary stenting [10]. The optimal initiation time and duration for CR following AMI has remained unclear [37]. An earlier study suggested that rehabilitation exercise may begin around 6 weeks after a cardiovascular event to ensure adequate medical stabilization and recovery [7]. Research in recent years has advanced the starting time for CR. A meta-analysis exploring the impact of exercise training on left ventricular remodeling after myocardial infarction suggests that early initiation time (around 1 week after AMI) and long-term exercise may confer the most significant benefits [11]. Some studies supported the significant benefits of community or family-based CR programs after PCI treatment and discharge for improving cardiac function and quality of life in patients with AMI. In line with the 2014 AHA/ACC guidelines for Non-ST-Elevation ACS, patients are generally advised to start aerobic exercise 1–2 weeks after undergoing PCI treatment and being discharged from the hospital [2]. A meta-analysis examining the effectiveness of home-based CR after PCI showed that it effectively enhanced cardiopulmonary function and reduced cardiovascular events [38].

Several meta-analyses examining the effectiveness of CR have consistently demonstrated that it can help reduce the incidence of cardiovascular events [16–18]. A large retrospective cohort study showed that in-hospital CR participation could significantly reduce the risk reduction of revascularisation, all-cause readmission and cardiac readmission among patients with PCI after AMI [39]. The ETICA trial has demonstrated that engaging in long-term moderate intensity exercise training after coronary angioplasty is safe [9]. Our study suggests that the initiation time and duration of CR had no significant impact on the occurrence of three types of cardiovascular events (arrhythmia, coronary artery restenosis and angina pectoris). Although the studies of the subgroups of all-cause mortality (ACM), MACE, and rehospitalization (RH) were not subjected to meta-regression analysis, we performed meta-analysis on the subgroups of ACM and MACE. The results showed small heterogeneity (ACM: I^2^ = 0.0%, *p* = 0.901; MACE: I^2^ = 45.0%, *p* = 0.162). Therefore, the timing of initiating CR within 1 month after PCI in AMI patients may not have a significant impact on the occurrence of cardiovascular events. According to the consensus of European Association of Cardiovascular Prevention and Rehabilitation, risk assessment is recommended for patients with ACS after PCI prior to starting physical activity. While some patients can safely start physical activity as early as the second day after PCI, those with large or complex myocardial injuries should gradually increase their physical activity level only after achieving clinical stability [40].

Cardiac color Doppler ultrasound and 6MWT are the main methods utilized for assessing cardiac function in patients with cardiovascular disease. Typically, CR and secondary prevention programs do not begin until at least 4–6 weeks after a patient is discharged from the hospital [8]. However, research suggests that patients who have had an uncomplicated myocardial infarction can benefit from earlier aerobic exercise training beginning as soon as 1 week after hospital discharge in order to achieve maximal anti-remodeling benefits. Additionally, it is recommended that these patients continue their aerobic exercise training for up to 6 months [11]. This viewpoint is also supported by the research conducted by Zheng H et al. and Jiang MH et al. [12, 13]. In our meta-regression analysis of observational studies, the starting time demonstrated a significant impact on the changes in LVEF. However, the meta-regression analysis of RCTs indicated that the initiation time and duration of CR had no significant impact on the changes in LVEF, LVEDV, and LVESV. Our main result is that during the one-month period following PCI, different initiation times and durations of CR did not have an impact on the changes in LVEF.

6MWT is a simple method for testing cardiopulmonary function. The result of meta-regression analysis did not reveal any significant association between changes in 6MWT and either initiation time or duration. 6MWT is self-paced, motivational factors could have significantly influenced performance and introduced variability across studies [41]. Therefore, meta-regression showed significant heterogeneity in the 6MWT results.

Due to the limited number of studies within the subgroups, it was not possible to conduct a meta-regression analysis on the time factors’ effects on heart rate, blood pressure, and blood lipid levels. The forest plot only reveals certain changing trends (Fig. 5). With increasing intervention time, both heart rate and LDL-C levels significantly decreased. TC decreased more significantly as the starting time advanced. But when the starting time was earlier, a smaller reduction was observed in systolic blood pressure (SBP). These conclusions did not reach statistical significance. Lowering blood pressure after a myocardial infarction may result in impaired perfusion of target organs [42]. The 2020 European Society of Cardiology guidelines for the management of acute coronary syndromes recommend that most individuals under 65 years of age, who are on blood pressure-lowering medications, should aim to lower their SBP to the range of 120–129 mmHg. For older patients aged 65 and above who are receiving these medications, it is generally recommended to target an SBP range of 130–139 mmHg [37]. The earlier CR is initiated, the potential benefits for the heart may be associated with a smaller decrease in blood pressure.

The included studies all started CR within 1 month after PCI. Short-term differences in the timing of rehabilitation initiation may not have a significant impact on the improvement of cardiac function. As a result, there is no substantial difference in the incidence of cardiovascular events due to variations in rehabilitation initiation time in the short term. It is important to recognize that CR is a comprehensive and long-term process, which can vary considerably in its specific implementation across studies due to factors such as patients’ foundational treatments, lifestyle habits and socioeconomic status. These variabilities contributed to the high heterogeneity observed in continuous variable data. The conflation of time-related factors within these variables may introduce interference when evaluating the influence of time on rehabilitation outcomes.

The prognosis of AMI is also influenced by the location and number of culprit blood vessels. Lesions in the left anterior descending (LAD) and left circumflex (LCx) were associated with higher mortality rates compared to lesions in the right coronary artery (RCA) [43]. A study demonstrated that patients with total occlusion ST-elevation myocardial infarction (STEMI) in LAD had a higher mortality rate during a 36-month follow-up period [44]. Patients with AMI and multivessel coronary disease have a poorer prognosis compared to individuals with single-vessel disease [45]. Only a small portion of the included studies reported the location of culprit blood vessels. Therefore, it was not possible to explore the correlation between the location of culprit blood vessels and the outcomes of CR. However, this factor should be considered as an important influencing factor in future research.

Overall, implementation of CR following PCI in patients with AMI is beneficial. However, the timing of initiating CR within 1 month after PCI in AMI patients and the duration of CR had no significant impact on the occurrence of arrhythmia, coronary artery restenosis, angina pectoris, and the changes in LVEF, LVEDV, LVESV and 6MWT. It indicates that it is feasible for patients with AMI to commence CR within 1 month after PCI and continue long-term CR, but the time factors which impact CR are intricate and further clinical research is still needed to determine the optimal initiation time and duration of CR.

## Limitation

First, because of a limited number of the studies, meta-regression analysis could not be conducted for certain subgroups. Further research is needed to evaluate these indicators in the future. Second, the included studies all started CR within 1 month after PCI. In the future, it may be necessary to compare the effects of initiation time across a wider range of starting times. Third, given that the majority of the studies included in this meta-analysis are from Asia, and the average age of the patients of the included studies is around 60 years old. There is a potential risk of selection bias in the overall pooled effect estimate. Fourth, the original data underwent a conversion process prior to consolidation, which may introduce some errors or inconsistencies in the final dataset.

## Conclusion

Implementation of CR following PCI in patients with AMI is beneficial. However, in AMI patients, there is no significant difference in the improvement of CR outcomes based on different CR starting times within 1 month after PCI or different durations of the CR programs. It indicates that it is feasible for patients with AMI to commence CR within 1 month after PCI and continue long-term CR, but the time factors which impact CR are intricate and further clinical research is still needed to determine the optimal initiation time and duration of CR.

### Supplementary Information

**Additional file 1.** S_Table 1 Retrieval strategy.

**Additional file 2.** S_Fig. 1 Quality assessment. (a) Risk of bias summary (RCT); (b) Risk of bias graph (RCT); (c) Risk of bias summary (observational study); (d) Risk of bias graph (observational study).

**Additional file 3.** S_Table 2 The results of meta-regression.

**Additional file 4.** S_Fig. 2 The funnel plots of the SMD for LVEF (RCTs).

**Additional file 5.** Data extraction.

**Additional file 6.** Detailed data of the figures.

## Data Availability

All data generated or analyzed during this study are included in this published article and its supplementary information files.

## Abbreviations

AMI: Acute myocardial infarction
PCI: Percutaneous coronary intervention
CR: Cardiac rehabilitation
ACM: All-cause mortality
ACS: Acute coronary syndrome
LVEF: Left ventricular ejection fraction
MACE: Major adverse cardiovascular events
RCT: Randomized controlled trial
6MWT: 6-minute walk test
RR: Risk ratio
CI: Confidence interval
SMD: Standardized mean difference
SD: Standard deviation
LVEDD: Left ventricular end-diastolic diameter
LVESD: Left ventricular end-systolic diameter
LVEDV: Left ventricular end-diastolic volume
LVESV: Left ventricular end-systolic volume
SBP: Systolic blood pressure
DBP: Diastolic blood pressure
TC: Total cholesterol
TG: Triglyceride
LDL-C: Low density lipoprotein cholesterol
RH: Rehospitalization
LAD: Left anterior descending
LCx: Left circumflex
RCA: Right coronary artery
STEMI: ST-elevation myocardial infarction
