# Supplementary material for: The impact of the time factors on the exercise-based cardiac rehabilitation outcomes of the patients with acute myocardial infarction after percutaneous coronary intervention: a systematic review and meta-analysis
Source: BMC Cardiovasc Disord. 2024 Jan 6;24:35. doi: 10.1186/s12872-023-03692-z (PMC10771662; doi:10.1186/s12872-023-03692-z)
Supplement: Supplementary file 1 — Additional file 1. S_Table 1 Retrieval strategy. [file 12872_2023_3692_MOESM1_ESM.docx]

| Database | Search Strategy | Result |
| --- | --- | --- |
| PubMed | ((((cardiac rehabilitation) OR (exercise)) OR (training)) AND (percutaneous coronary intervention)) AND (acute myocardial infarction) | 1432 |
| Embase | ('cardiac rehabilitation':ab,ti OR exercise:ab,ti OR training:ab,ti) AND 'percutaneous coronary intervention':ab,ti AND 'acute myocardial infarction':ab,ti AND [<1966-2023]/py | 314 |
| Cochrane Library | (((cardiac rehabilitation) OR (exercise)) OR (training)) AND (percutaneous coronary intervention) AND (acute myocardial infarction) | 285 |
| Web of Science | ((((ALL=(cardiac rehabilitation)) OR ALL=(exercise)) OR ALL=(training)) AND ALL=(percutaneous coronary intervention)) AND ALL=(acute myocardial infarction) | 850 |
| Google scholar | (intitle:"cardiac rehabilitation" OR intitle:"exercise" OR intitle:"training") AND (intitle:"percutaneous coronary intervention") AND (intitle:"acute myocardial infarction") | 56 |

S_Table 1 Retrieval strategy
