# Supplementary material for: The impact of the time factors on the exercise-based cardiac rehabilitation outcomes of the patients with acute myocardial infarction after percutaneous coronary intervention: a systematic review and meta-analysis
Source: BMC Cardiovasc Disord. 2024 Jan 6;24:35. doi: 10.1186/s12872-023-03692-z (PMC10771662; doi:10.1186/s12872-023-03692-z)
Supplement: Supplementary file 2 — Additional file 2. S_Fig. 1 Quality assessment. (a) Risk of bias summary (RCT); (b) Risk of bias graph (RCT); (c) Risk of bias summary (observational study); (d) Risk of bias graph (observational study). [file 12872_2023_3692_MOESM2_ESM.pdf]

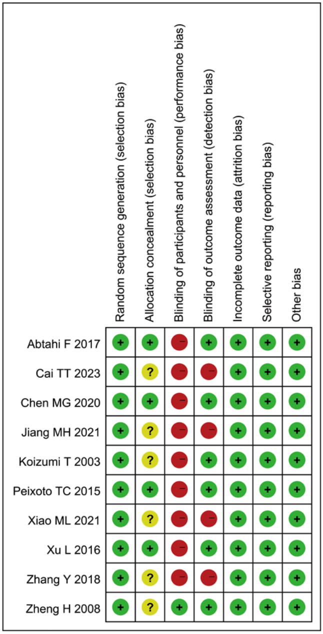

(a) Risk of bias summary (RCT)

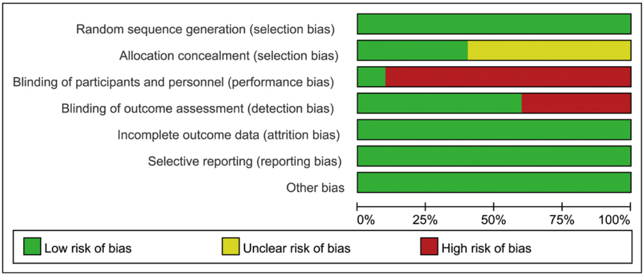

(b) Risk of bias graph (RCT)

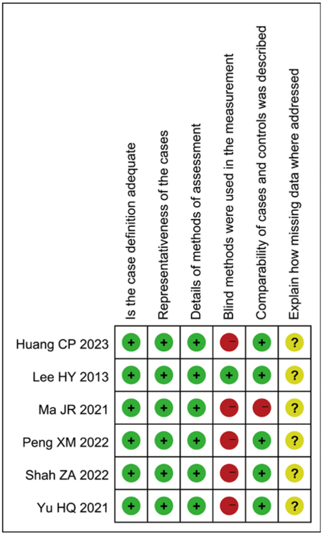

(c) Risk of bias summary (Observational Study)

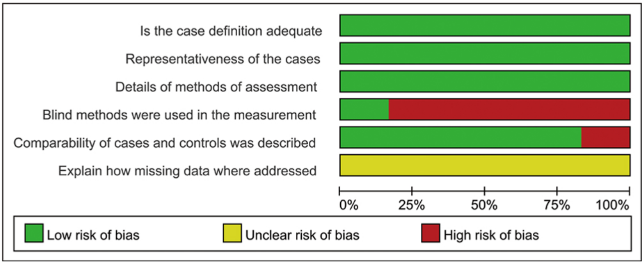

(d) Risk of bias graph (Observational Study)
