# Supplementary material for: The impact of the time factors on the exercise-based cardiac rehabilitation outcomes of the patients with acute myocardial infarction after percutaneous coronary intervention: a systematic review and meta-analysis
Source: BMC Cardiovasc Disord. 2024 Jan 6;24:35. doi: 10.1186/s12872-023-03692-z (PMC10771662; doi:10.1186/s12872-023-03692-z)
Supplement: Supplementary file 3 — Additional file 3. S_Table 2 The results of meta-regression. [file 12872_2023_3692_MOESM3_ESM.docx]

S_Table 2 The results of meta-regression

**Arrhythmia (Observational Study)**

|  | Estimate | SE | Z value | P value | 95% CI |
| --- | --- | --- | --- | --- | --- |
| Intrcpt | 0.2212 | 0.6345 | 0.3487 | 0.7273 | [-1.0223, 1.4648] |
| Starting time | -0.0071 | 0.1524 | -0.0467 | 0.9627 | [-0.3058, 0.2916] |
| Intervention time | 0.0157 | 0.0469 | 0.3348 | 0.7378 | [-0.0761, 0.1075] |

tau^2^ = 0, I^2^ = 0.00%, R^2^ = 0.00%

QE = 0.0482 (df = 1, p = 0.8263), QM = 0.1130 (df = 2, p = 0.9450)

**Coronary artery restenosis (Observational Study)**

|  | Estimate | SE | Z value | P value | 95% CI |
| --- | --- | --- | --- | --- | --- |
| Intrcpt | 0.5168 | 0.7063 | 0.7318 | 0.4643 | [-0.8674, 1.9011] |
| Starting time | 0.0662 | 0.1967 | 0.3365 | 0.7365 | [-0.3193, 0.4517] |
| Intervention time | -0.0127 | 0.0326 | -0.3908 | 0.6959 | [-0.0766, 0.0511] |

tau^2^ = 0, I^2^ = 0.00%, R^2^ = 0.00%

QE = 0.0254 (df = 2, p = 0.9874), QM = 0.2090 (df = 2, p = 0.9008)

**Angina pectoris (Observational Study)**

|  | Estimate | SE | Z value | P value | 95% CI |
| --- | --- | --- | --- | --- | --- |
| Intrcpt | 0.2933 | 0.5514 | 0.5320 | 0.5947 | [-0.7873, 1.3740] |
| Starting time | 0.0260 | 0.1606 | 0.1622 | 0.8712 | [-0.2886, 0.3407] |
| Intervention time | -0.0028 | 0.0504 | -0.0560 | 0.9553 | [-0.1017, 0.0960] |

tau^2^ = 0, I^2^ = 0.00%, R^2^ = 0.00%

QE = 0.0042 (df = 1, p = 0.9484), QM = 0.0266 (df = 2, p = 0.9868)

**LVEF (RCT)**

|  | Estimate | SE | Z value | P value | 95% CI |
| --- | --- | --- | --- | --- | --- |
| Intrcpt | 0.2480 | 0.3147 | 0.7881 | 0.4307 | [-0.3688, 0.8649] |
| Starting time | 0.1604 | 0.1061 | 1.5125 | 0.1304 | [-0.0475, 0.3683] |
| Intervention time | 0.0168 | 0.0117 | 1.4440 | 0.1487 | [-0.0060, 0.0397] |

tau^2^ = 0.1479, I^2^ = 72.87%, R^2^ = 24. 97%

QE = 27.8681 (df = 8, p = 0.0005), QM = 3.6262 (df = 2, p = 0.1631)

**LVEF (Observational Study)**

|  | Estimate | SE | Z value | P value | 95% CI |
| --- | --- | --- | --- | --- | --- |
| Intrcpt | 1.8178 | 0.5044 | 3.6043 | 0.0003 | [0.8293, 2.8063] |
| Starting time | -0.3319 | 0.0928 | -3.5785 | 0.0003 | [-0.5137, -0.1501] |
| Intervention time | -0.0306 | 0.0226 | -1.3545 | 0.1756 | [-0.0748, 0.0137] |

tau^2^ = 0.0304, I^2^ = 51.66%, R^2^ = 88.92%

QE = 2.0688 (df = 1, p = 0.1503), QM = 12.8298 (df = 2, p = 0.0016)

**LVEDV (RCT)**

|  | Estimate | SE | Z value | P value | 95% CI |
| --- | --- | --- | --- | --- | --- |
| Intrcpt | 0.3618 | 0.5211 | 0.6942 | 0.4875 | [-0.6596, 1.3832] |
| Starting time | -0.1912 | 0.5574 | -0.3430 | 0.7316 | [-1.2836, 0.9012] |
| Intervention time | -0.0330 | 0.0235 | -1.4040 | 0.1603 | [-0.0791, 0.0131] |

tau^2^ = 0.0675, I^2^ = 61.57%, R^2^ = 12.03%

QE = 2.6024 (df = 1, p = 0.1067), QM = 2.1517 (df = 2, p = 0.3410)

**LVESV (RCT)**

|  | Estimate | SE | Z value | P value | 95% CI |
| --- | --- | --- | --- | --- | --- |
| Intrcpt | -0.1723 | 0.3796 | -0.4540 | 0.6498 | [-0.9163, 0.5717] |
| Starting time | -0.3005 | 0.4104 | -0.7323 | 0.4640 | [-1.1050, 0.5039] |
| Intervention time | 0.0148 | 0.0164 | 0.9001 | 0.3681 | [-0.0174, 0.0470] |

tau^2^ = 0, I^2^ = 0.00%, R^2^ = 100.00%

QE = 0.2913 (df = 1, p = 0.5894), QM = 2.8712 (df = 2, p = 0.2380)

**6MWT (RCT)**

|  | Estimate | SE | Z value | P value | 95% CI |
| --- | --- | --- | --- | --- | --- |
| Intrcpt | 1.0187 | 0.4140 | 2.4605 | 0.0139 | [0.2072, 1.8302] |
| Starting time | -0.1075 | 0.1476 | -0.7283 | 0.4665 | [-0.3967, 0.1818] |
| Intervention time | 0.0193 | 0.0123 | 1.5700 | 0.1164 | [-0.0048, 0.0435] |

tau^2^ = 0.1404, I^2^ = 76.82%, R^2^ = 32.37%

QE = 7.7915 (df = 2, p = 0.0203), QM = 3.4727 (df = 2, p = 0.1762)
