# Supplementary figures and images for: The impact of the time factors on the exercise-based cardiac rehabilitation outcomes of the patients with acute myocardial infarction after percutaneous coronary intervention: a systematic review and meta-analysis
Source: BMC Cardiovasc Disord. 2024 Jan 6;24:35. doi: 10.1186/s12872-023-03692-z (PMC10771662; doi:10.1186/s12872-023-03692-z)

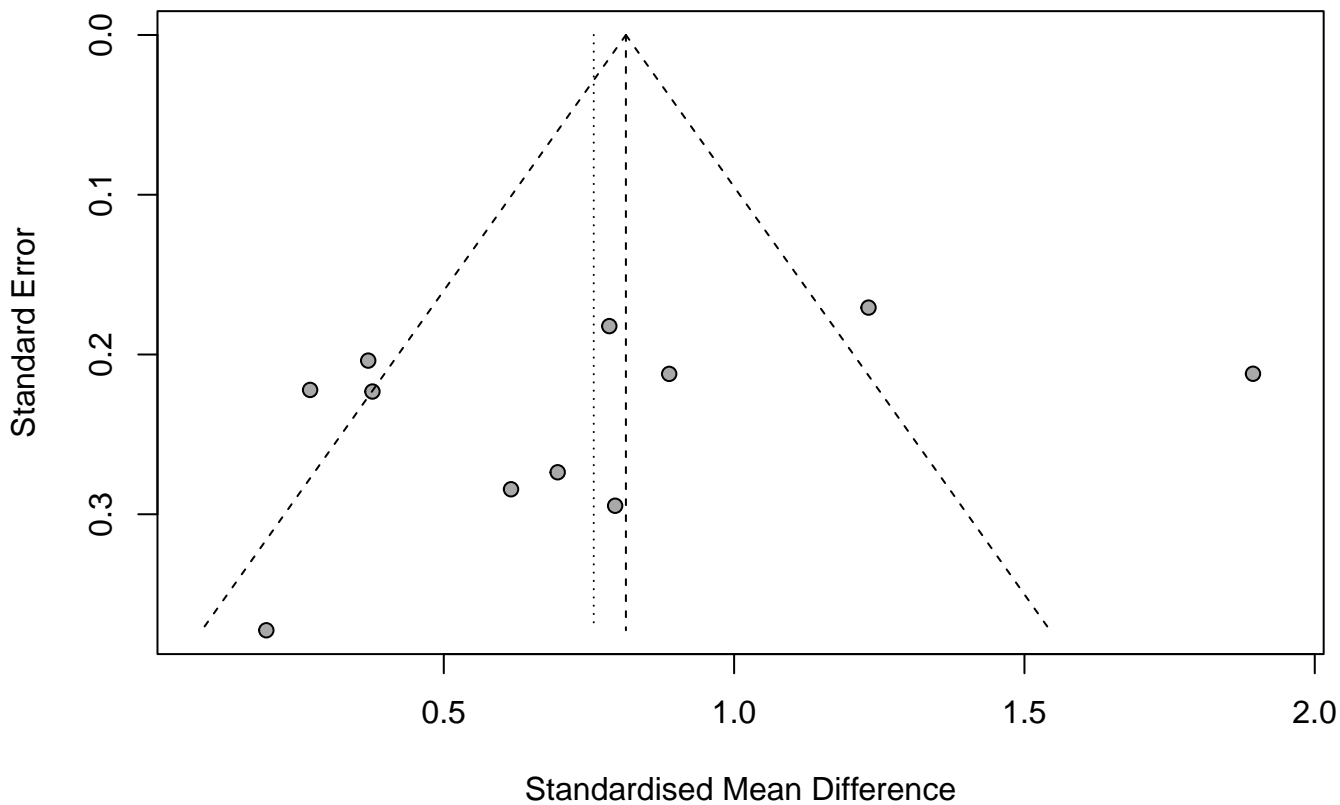

Supplement: Supplementary file 4 — Additional file 4. S_Fig. 2 The funnel plots of the SMD for LVEF (RCTs). [file 12872_2023_3692_MOESM4_ESM.pdf]
